# Surgical predictors of acute postoperative pain after hip arthroscopy

**DOI:** 10.1186/s12871-015-0077-x

**Published:** 2015-07-02

**Authors:** Chong Oon Tan, Yew Ming Chong, Phong Tran, Laurence Weinberg, William Howard

**Affiliations:** 1 Department of Anaesthesia, The Austin Hospital, 145 Studley Rd, Heidelberg, VIC 3084 Australia; 2 The University of Melbourne, Melbourne, VIC 3010 Australia; 3 Department of Surgery, St. Vincent’s Hospital Melbourne, Victoria Pde, Fitzroy, VIC Australia; 4 Department of Orthopaedics, Western Health, Gordon St, Footscray, VIC 3011 Australia

**Keywords:** Hip arthroscopy, Postoperative pain, Morphine, Numerical rating scale, Regional anaesthesia, Arthroscopic fluid infusion pressure, Labral repair, Femoral chondro-osteoectomy

## Abstract

**Background:**

Pain following hip arthroscopy is highly variable and can be severe. Little published data exists demonstrating reliable predictors of significant pain after hip arthroscopy. The aim of this study was to identify influence of intraoperative factors (arthroscopic fluid infusion pressure, operative type) on the severity of postoperative pain.

**Methods:**

A retrospective review of 131 patients who had received a variety of arthroscopic hip interventions was performed. A standardized anaesthetic technique was used on all patients and postoperative pain was analysed using recovery pain severity outcomes and analgesic use. A multivariate logistic regression analysis was performed on intraoperative factors including patient age, sex and BMI, arthroscopic infusion pressures (40 vs 80 mm Hg), amount of fluid used, length of surgery and types of arthroscopic interventions performed. Thirty six patients were also prospectively examined to determine arthroscopic fluid infusion rates for 40 and 80 mm Hg infusion pressures.

**Results:**

Use of a higher infusion pressure of 80 mm Hg was strongly associated with all pain severity endpoints (OR 2.8 – 8.2). Other significant factors included hip arthroscopy that involved femoral chondro-ostectomy (OR 5.8) and labral repair (OR 7.5). Length of surgery and total amount of infusion fluid used were not associated with increased pain.

**Conclusions:**

80 mm Hg arthroscopic infusion pressures, femoral chondro-osteoectomy and labral repair are strongly associated with significant postoperative pain, whereas intraoperative infusion volumes or surgical duration are not. Identification of these predictors in individual patients may guide clinical practice regarding the choice of more invasive regional analgesia options. The use of 40 mm Hg arthroscopic infusion pressures will assist in reducing postoperative pain.

## Background

Pain following hip arthroscopy is highly variable and in some cases can be severe, with high patient Numerical Rating Scale (NRS) pain scores up to 8/10 and associated high postoperative opioid requirements [1]. An analgesic management approach based on systemic analgesia alone is limited by opioid side effects, some of which are more frequent in the predominantly younger patient population receiving this procedure. Other analgesic techniques that may be used include regional analgesia, neuraxial local analgesia, and intrathecal opioids, but carry with them their own potentially serious side effects [2–5]. Although psychological distress is known to influence postoperative pain in this context [6], there is currently no other data in the literature identifying surgical risk factors of severe postoperative pain in hip arthroscopy. We hypothesized that use of a lower arthroscopic infusion pressure (40 mm Hg) would minimise periarticular soft tissue swelling, and hence reduce acute postoperative pain. We also hypothesized that clinical and intraoperative factors such as patient demographics, total arthroscopic fluid volumes infused and the particular surgical interventions performed were related to the degree of patient’s postoperative pain. Identification of reliable predictors of severe postoperative pain would allow directed use of more invasive analgesia options, justifying the rare but potentially serious risks of such interventions.

## Methods

We performed a retrospective study (Austin Health Office for Research, Human Research & Ethics Committee Approval # LNR/14/Austin/2) of 140 consecutive patients in 2013 who had undergone therapeutic hip arthroscopy by a single surgeon and anaesthetist with combined subspecialty experience in the procedure of more than 8 years. From May 2013, the treating surgeon changed the routine arthroscopic infusion pressures used from 80 mm Hg to 40 mm Hg for all hip arthroscopies. Arthroscopic infusion pressures were kept the same throughout the entirety of each patient’s procedure. Inclusion criteria were all patients undergoing hip arthroscopy who had received the same standardised anaesthetic and postoperative pain regime. Patients who had contraindications to any component of the standard anaesthetic technique such as medication allergy or opioid tolerance were excluded from the study.

All patients were ASA (American Society of Anesthesiologists Preoperative Assessment Score) 1 or 2 and were elective admissions planned for overnight stay. Each patient received spontaneous ventilation volatile agent general anaesthesia with a laryngeal mask airway and sevoflurane. All patients also received 1 g IV paracetamol, 1.5 mg/kg IV tramadol and 40 mg IV parecoxib analgesia intraoperatively, as well as pre-emptive antiemesis of 8 mg IV dexamethasone and 1 mg IV granisetron. Alfentanil was titrated intraoperatively in 250mcg boluses to a respiratory rate of 10–12 at the end of the procedure. Patients in recovery received further IV morphine boluses of 1–2 mg 5 minutely titrated primarily to (1) patient’s satisfaction with their level of analgesia such that patients declined further opioid administration; (2) lack of signs of impending opioid narcosis (delayed eye opening in response to verbal stimuli and/ or respiratory rate 12 or below) and (3) patient’s reported NRS score of 6 or below. All opioid doses were converted to IV morphine equivalents as per MacIntyre [7].

All patients underwent hip arthroscopy in the lateral position under general anaesthesia. Under fluoroscopic guidance, traction was applied (McCarthy distractor; Innomed, Savannah, GA) and the instruments were placed through mid-trochanteric and anterior paratrochanteric portals using the Arthrex Continuous Wave 3 Arthroscopy Pump (Arthrex, Naples, FL, USA). At the end of the surgical procedure, the joint was lavaged and injected with ropivacaine 200 mg and morphine 5 mg.

Pain outcomes assessed were the highest and lowest NRS scores in recovery, and the total intraoperative and recovery room opioid requirements. These endpoints were used as markers of significant postoperative pain as (a) the complex relationship between pain scores and opioid use requires both endpoints to be measured and (b) the highest pain score in recovery is a marker of initial pain following a standardised intraoperative analgesic regimen, whereas the lowest pain score reflects the degree of residual pain after maximum safe doses of opioid analgesia have been administered to the patient. Patient demographics and specific surgical interventions were also recorded (Table 1). Nine patients were excluded from analysis as they had not received the standard multimodal analgesia regime, eg. employment of a fascia iliaca block, ketamine analgesia, prior opioid tolerance, or contraindications to any component of the standard anaesthetic technique. Operative time was defined as time from application of traction to placement of final suture.

**Table 1:** Patient demographics and perioperative parameters^a^

Age (years)	34 (18 – 73)
Sex (M: F)	89 (68 %) : 42 (32 %)
Weight (kg)	82 (45 – 135)
Arthroscopic infusion pressures (40mmhg: 80mmhg)	62 (47 %) : 69 (53 %)
Average total arthroscopic fluid volume infused (L)	14.6 (3.8 – 47.7)
Average total perioperative opioid requirements (IV morphine equivalents, mg)	20 (5 – 40)
Duration of surgery (mins)	54 (18 – 154)
Operation types^b^:	
Arthroscopic debridement of ligamentum teres	30 (57 %)
Femoral chondro-osteectomy	86 (70 %)
Acetabular osteoectomy	16 (12 %)
Labral repair	82 (63 %)
Synovectomy	64 (49 %)
Arthroscopic chondroplasty	18 (14 %)

a) Data are presented as mean (range) or number (proportion)

b) Many patients received more than one type of surgical procedure

As total arthroscopic fluid volumes infused are not charted routinely, we also prospectively observed the fluid volumes used per unit time in a series of 36 patients, half of which had received infusion pressures of 80 mm Hg and the other half who had received 40 mm Hg throughout the entirety of their procedures. Once mean point estimates of ml/ min in each prospective group had been calculated, these were used to derive fluid volumes given/ min in our study sample.

### Statistical analyses

Statistical analysis was performed using SPSS version 21 (IBM, New York, USA). Association between continuous variables was analysed by linear regression and correlation. Prediction bands were calculated around the regression lines to estimate dependent variable outcomes for a given value of any independent variable. Patient characteristics of age, sex and BMI as well as likely operative predictors for severe postoperative pain were analysed by a multivariate logistic regression model against outcomes of highest recovery room NRS >4, lowest recovery room NRS <2 and intraoperative/ recovery room IV morphine requirements of over 0.15 mg/ kg. NRS outcome cutoffs were selected based on accepted definitions of “moderate-to-severe” and “mild” postoperative pain [8], and the IV morphine requirement cutoff chosen as 1 SD (standard deviation) from mean postoperative morphine requirements in hip arthroscopy as established by Morgenthaler [9]. *P* values of <0.05 were considered statistically significant. The total number of variables included in the regression model did not exceed the conservative ratio of 1 variable per 10 samples [10].

## Results

The demographics and perioperative information of the 131 patients included in the study are presented in Table 1.

**Fig. 1:**
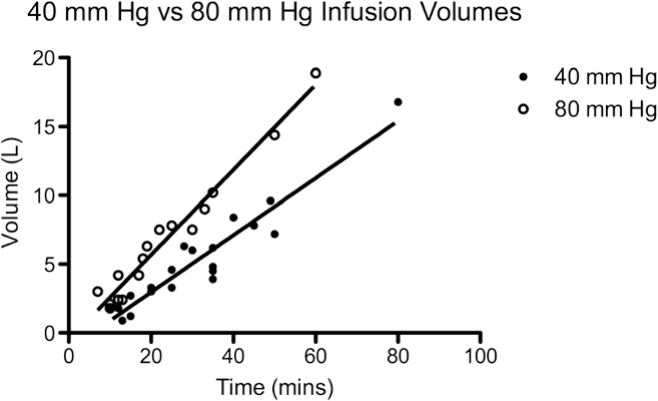
Linear regression of infusion volumes per unit time, 40 mm hg versus 80 mm Hg infusion pressure

Duration of arthroscopic infusion was strongly correlated with total infusion volumes (R^2^ 0.91–0.97 at *p* < 0.0001 for 40 and 80 mm Hg infusion pressures respectively, Fig. 1).

**Fig. 2:**
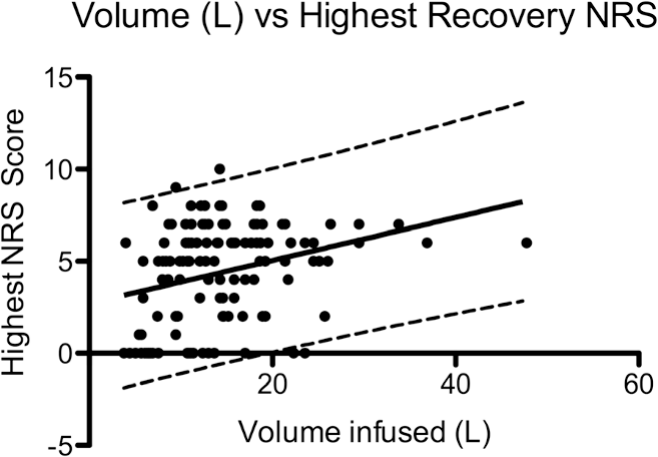
Linear regression of total intraoperative arthroscopic fluid volumes versus recovery room highest NRS score

**Fig. 3:**
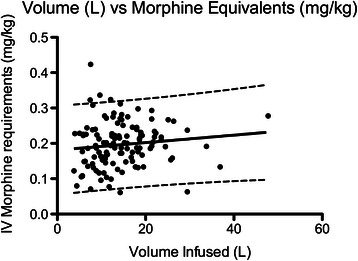
Linear regression of total intraoperative arthroscopic fluid volumes versus intraoperative & recovery room morphine requirements

Patients who underwent arthroscopy with 80 mm Hg infusion pressure received arthroscopic fluid volumes at a rate of 300 ml/min (95 % CI 290–310 ml/min) into the hip joint and surrounding tissues compared with 210 ml/min for those whose infusion pressure was 40 mm Hg (200–220 ml/min, R^2^ > 0.9, *p* < 0.0001). However, there was only minor correlation between total volumes infused and the pain severity outcomes of NRS scores and IV morphine requirements (largest Pearson’s R <0.36, Table 2, Figs. 2,3). Statistical significance was achieved for these dependent variables, however the prediction bands around the regression lines were broad (Table 2).

**Table 2:** Correlation analysis between total arthroscopic infusion volumes and pain severity outcomes

Pain severity outcome measure	Pearson’s R	*P*-value	Regression slope (parameter/ L)	Prediction band range
Highest recovery NRS score (units/ 10)	0.36	0.0004	0.12	2 – 10
Lowest recovery NRS (units/ 10)	0.31	<0.0001	0.10	0 – 7
Perioperative IV morphine equivalents (mg/ kg)	0.12	0.19	0.001	0.06 – 0.32

High pressure arthroscopic fluid infusion was associated the most strongly with all pain severity endpoints (Odds Ratio [OR] 2.8 – 8.2, Table 3). Procedures that involved femoral chondro-osteoectomy (OR 5.8) and labral repair (OR 7.5) were significantly associated with perioperative morphine use of >0.15 mg/kg. No other intraoperative factors (total volume of arthroscopic fluid infused, duration of surgery, performance of synovectomy, acetebular ostectomy, chondroplasty and/ or debridement of ligamentum teres) nor patient factors (age, sex, BMI) achieved statistical significance in the multivariate logistic regression model.

**Table 3:** Intraoperative predictors significantly associated with adverse recovery pain outcomes

Intraoperative predictor	OR (Odds ratio)	*P*-value	Pain severity outcome measure
Use of 80 mm Hg arthroscopic fluid infusion pressure	2.8	0.01	Highest recovery NRS pain score >4
	8.2	0.01	Perioperative IV morphine use >0.15 mg/kg
Femoral chondro-ostectomy	5.8	0.02	
Labral repair	7.5	0.01	

## Discussion

Acute pain management after hip arthroscopy continues to be a challenge for the perioperative team. The acute postoperative pain from the procedure can be difficult to predict; from our experience we have found that with primary use of systemic analgesia alone, 3 % of patients required rescue regional analgesia and 1.5 % required postoperative ketamine in the recovery room. This contrasts with some patients who require no or minimal recovery opioid and request discharge on the day of surgery. Some practitioners advocate routine use of pre-emptive regional or neuraxial analgesia to ensure ideal pain relief on emergence from anaesthesia. Whilst this approach guarantees a smooth recovery for all patients, it may be exposing some patients unnecessarily to permanent neurological injury, risk of delayed discharge, late mobilisation [11] and falls [12]. We present findings from 131 patients who had undergone hip arthroscopy that may assist in predicting those who may benefit most from these invasive analgesic techniques.

Our results show that several intraoperative factors have profound influence on the likelihood of patients suffering significant postoperative pain, whilst other clinical predictors bore no relationship to pain severity. The precise causes of severe pain following hip arthroscopy are unknown. Our results suggest however that it is the pressure at which the fluid is infused rather than the total volume that has more bearing on postoperative pain. We propose that it is the higher infusion pressure which distends the joint capsule and results in an increased net flux of fluid into the periarticular soft tissues. Use of 80 mm Hg infusion pressures was the strongest clinical predictor (highest OR of 8.2) and achieved statistical significance across all pain severity outcome measures, whereas operative intervention types influenced perioperative IV morphine use alone. An 80 mm Hg infusion pressure is frequently used amongst hip arthroscopy specialists as the commonly held belief is that a high pressure allows superior surgical access, however there is no evidence that the use of this infusion pressure confers any advantage. Indeed, the surgical author found that less periarticular soft tissue swelling developed during long cases where 40 mm Hg was used, resulting in less restriction of arthroscopic instrument mobility within the joint.

Although volumes infused vs. highest and lowest recovery pain scores achieved statistical significance with regards to correlation, the strength of correlation was weak (highest pearson’s R = 0.36) and the prediction bands around the regression lines too broad to be considered useful in clinical practice (NRS range of +/- 4 for a given amount of fluid infused). Correlation between arthroscopic infusion volumes and perioperative morphine requirements did not reach statistical significance; this result may be explained by the confounding effects of the routinely co-administered analgesics (intra-articular local anaesthetic and opioid, non-steroidal anti-inflammatory and tramadol) as well as the common observation that in situations of intense postoperative pain and escalating opioid dosing, early signs of sedation and respiratory depression contraindicate further opioid administration even though patient reported NRS scores may remain elevated. Surgical duration and hence total volumes infused also did not achieve statistical significance in the multivariate logistic regression model for independent predictors of analgesia outcomes. It is possible that the ratio of amount of fluid absorbed into soft tissue as opposed to that lost out of surgical access portals is affected by arthroscopic infusion pressure.

Although the findings of Baker et al [1] suggested that the type and degree of tissue damage in hip arthroscopy are not related to postoperative pain, our results are significantly different. We found in our study that this applied to interventions involving a greater amount of bone resection (femoral chondro-osteoectomy) but not acetabular osteoectomy or chondroplasty, which require less bone resection. Labral repair was also associated with significant increase in likelihood of postoperative pain (OR 7.5), however other soft tissue interventions with a lower degree of tissue trauma (synovectomy, debridement and chondroplasty) were not.

Our study was limited by its retrospective design and moderate sample size. The use of multivariate logistic regression modelling however is robust in the face of non-normally distributed data and lack of randomisation; nor are direct cohort comparisons required. Although our study design was based on a single centre sample, it has ensured that a standardised approach to anaesthesia and surgical technique had been applied. The use of derived values for total arthroscopic fluid volumes infused may introduce some degree of error, but it is clear from our results that infusion volumes at 80 versus 40 mm Hg are significantly different (*p* < 0.0001) and with a precise point estimate (95 % CI +/- 10 ml/ min). Intra-articular fluid pressures can potentially vary significantly despite arthroscopic pump settings and continuous measurements performed by arthroscopic pumps are made by an internal systems pressure gauge, and hence may not reflect true intra-articular pressure. Future studies using continuous direct measurement of intra-articular fluid pressures during surgery would be warranted.

Qualitatively we observed that the use of lower fluid infusion pressures did not compromise surgical view quality and access. Indeed, the use of 80 mm Hg infusion pressure led to decreased mobility of arthroscopic instruments due to a greater degree of soft tissue swelling around port access sites.

## Conclusions

A higher arthroscopic fluid infusion pressure of 80 mm Hg is strongly associated with significant postoperative pain, whereas total infusion fluid volumes and surgical duration are not. Arthroscopic interventions involving extensive bony resection and soft tissue repair – femoral chondro-ostectomy and labral repair – also have a significant influence on postoperative pain. Identification of these predictors in individual patients may guide clinical practice regarding the choice of more invasive regional analgesia options. If a lower arthroscopic infusion pressure of 40 mm Hg does not impair the surgeons view and access, we recommend its use to reduce the risk of patients developing severe postoperative pain.

## Consent

Written informed consent was waived by the Austin Health Human Research & Ethics Department for the purposes of this study.
